# Association of glutamate metabotropic receptor polymorphisms and sensorineural hearing loss in adults of different age groups^☆^

**DOI:** 10.1016/j.bjorl.2018.04.007

**Published:** 2018-05-18

**Authors:** Sanlin Xie, Jianzhong Li, Wentao Wang, Xianming Chen

**Affiliations:** Fuzhou General Hospital, Department of Otolaryngology-Head and Neck Surgery, Fujian Sheng, China

**Keywords:** Sensorineural hearing loss, Glutamate metabotropic receptor 7, Single nucleotide polymorphism, Perda auditiva neurossensorial, Receptor metabotrópico de glutamato 7, Polimorfismo de nucleotídeo único

## Abstract

**Introduction:**

Sensorineural hearing loss is a common challenge all over the world, including a section of the young population. While there have been many published reports associating glutamate metabotropic receptor 7 with sensorineural hearing loss, there is no report, till date, about the association of glutamate metabotropic receptor 7 polymorphisms with sensorineural hearing loss at different ages.

**Objective:**

To test the association between the single nucleotide polymorphisms rs11928865 and rs11920109 of the glutamate metabotropic receptor 7 with sensorineural hearing loss in adults of different age groups.

**Methods:**

A total of 1661 subjects were studied. The individuals aged between 30 and 50, and between 51 and 70 years with sensorineural hearing loss comprised group A and group B, respectively. Individuals aged between 30 and 50; and between 51 and 70 years without hearing loss comprised control groups C and D, respectively. The MassARRAY method was used to analyze the genotypes.

**Results:**

The difference in genotypes for the glutamate metabotropic receptor 7 rs11928865 single nucleotide polymorphism between patients in the groups B and D was statistically significant (*p* = 0.018). The distribution frequencies of genotypes in patients that were aged between 30 and 50 years were not significantly different. The difference in genotypes for the rs11920109 single nucleotide polymorphism between the sensorineural hearing loss groups and control groups showed no statistical significance.

**Conclusion:**

The rs11928865 single nucleotide polymorphism was associated with the susceptibility to hearing loss in patients in group B but not with those in group A.

## Resumo

**Introdução:**

A perda auditiva neurossensorial é um desafio comum no mundo todo, inclui uma parte da população jovem. Embora haja muitos relatos que associem o gene do receptor metabotrópico de glutamato 7 com perda auditiva neurossensorial, não há relato, até a presente data, sobre a associação de polimorfismos do receptor metabotrópico de glutamato 7 com perda auditiva neurossensorial em diferentes faixas etárias.

**Objetivo:**

Testar a associação entre os polimorfismos de nucleotídeo único, rs11928865 e rs11920109 do receptor metabotrópico de glutamato 7 e perda auditiva neurossensorial em adultos de diferentes faixas etárias.

**Método:**

Um total de 1661 indivíduos foram estudados. Os indivíduos com idade entre 30 e 50 anos e entre 51 e 70 anos com perda auditiva neurossensorial constituíram o grupo A e o grupo B, respectivamente. Indivíduos com idade entre 30 e 50 anos; e entre 51 e 70 anos sem perda auditiva foram os grupos controle C e D, respectivamente. O método MassARRAY foi utilizado para analisar os genótipos.

**Resultados:**

A diferença nos genótipos para o polimorfismo de nucleotídeo único rs11928865 do gene receptor metabotrópico de glutamato 7 entre os pacientes dos Grupos B e D foi estatisticamente significante (*p* = 0,018). As frequências de distribuição dos genótipos nos pacientes entre 30 e 50 anos não foram significantemente diferentes. A diferença nos genótipos para o polimorfismo de nucleotídeo único rs11920109 entre os grupos com perda auditiva neurossensorial e os grupos controle não mostrou significância estatística.

**Conclusão:**

O polimorfismo de nucleotídeo único rs11928865 foi associado à suscetibilidade para perda auditiva em pacientes do grupo B, mas não àqueles do grupo A.

## Introduction

Single nucleotide polymorphisms (SNPs) may cause changes in the quality or quantity of protein expression, which may be related to the occurrence of certain diseases or the susceptibility to diseases. Glutamate is an important neurotransmitter of the auditory system. The protein product of the glutamate metabotropic receptor 7 gene (GRM7), mGluR7, is widely distributed in the inner and outer hair cells and spiral ganglion nerve cell. Sensorineural hearing loss (SHL) is a common challenge all over the world, including a section of the young population. There have been many published reports associating GRM7 with SHL. Friedman et al. have associated the gene GRM7 with susceptibility to SHL for individuals from European countries.^1^ The presence of GRM7 SNP correlates significantly with SHL in elderly Chinese Han men, and different types of hearing loss present different gene polymorphisms.^2^ However, the SHL subjects in these studies were all over 50 years of age. Interestingly, there is no report, till date, about the association of GRM7 polymorphisms with SHL at different ages. Whether younger subjects with SHL also feature an association GRM7 SNPs is unclear.

Here, we selected a group of SHL patients between 30–50 and 51–70 years-of-age and corresponding control groups for a genetic analysis of GRM7. We compared the susceptibility to SHL in the patients of these different age groups with GRM7 SNPs.

## Methods

### Subjects

A total of 1661 Han Chinese volunteers participated in this study. They were all 30–70 years-of-age. They visited our hospital between January 2013 and January 2017. The study was approved by the Ethics Committee of Hospital and written informed consent was obtained from each subject; the ethics committee's approval number was 2015003. A total of 467 subjects, aged between 30 and 50 years, including 236 men and 231 women with a mean age of 40.54 ± 5.32 years, with SHL comprised group A. A total of 278 individuals, aged between 30 and 50 years, including 147 men and 131 women with a mean age of 40.23 ± 4.99 years, without SHL comprised control group C. A total of 439 subjects, aged between 51 and 70 years, including 208 men and 231 women with a mean age of 62.02 ± 3.44 years, with SHL comprised group B. Finally, 477 individuals, aged between 51 and 70 years, including 257 men and 220 women with a mean age of 61.08 ± 4.31 years, without SHL comprised control group D.

### Clinical evaluation and audiological measurement

All participants underwent a routine annual health check-up that included a short questionnaire of demographic data, systemic disease history, personal life history for habits such as smoking and drinking, chest X-ray electrocardiography, and biochemical profiling of blood samples.

All subjects were analyzed by pure tone audiometry, tympanometry, and examination of the auditory brainstem response (ABR). Audiometry was done using a model GSI-61 pure tone audiometer (Grason-Stadler, Inc., Madison WI, USA). Tympanometry used the GSI-Tymp star II (Grason-Stadler, Inc.). The auditory brainstem response was measured using the Smart EP (Intelligent Hearing Systems, Miami, FL, USA). The pure tone threshold average (PTA) of the patients’ air frequency at 0.5 kHz, 1 kHz, 2 kHz, and 4 kHz were calculated for each individual, and then individuals were grouped according to the PTA of the ears. The decrease in hearing in the experimental group reached beyond the 26 dB hearing level (HL) of two or more consecutive drops in frequency. The subjects were examined with ABR, and the peak latency of I, III, and V wave, and the interpeak latency of I–V wave were determined, and interlateral latency difference of the peak latency and interpeak latency were compared. When peak latency of V wave extended longer than 6.1 ms; or wave III and wave V could not be induced; or the I–V wave interpeak latency lengthening was greater than or equal to 4.0 ms; or the interlateral latency difference with V wave was greater than 0.4 ms, and those who were considered positive for postcochlear lesions, those who meet these conditions will not be included in the research. Subjects were excluded if they had conductive deafness, noise-induced deafness, and deafness caused by other diseases such as drug-induced deafness, sudden deafness, Meniere's disease, otosclerosis, adhesive otitis, acoustic neuroma, and autoimmune deafness. Additionally, subjects were also excluded if they had systemic diseases including diabetes, hypertension, heart disease, hyperlipidemia, chronic kidney disease, chronic obstructive pulmonary disease, and alcohol abuse. Among the candidates who met these conditions, subjects who had progressive loss of hearing after the age of 50 years were categorized in Group B, whereas subjects who had progressive hearing loss after 29 years of age were allotted to Group A.

### Sequenom MassARRAY SNP analysis tests

Based on prior studies, rs11928865 and rs11920109 were selected as the GRM7 SNPs relevant to age-related SHL.^1^, ^2^ Genotyping was performed using polymerase chain reaction (PCR) amplification and MassARRAY-specific SNP analyses. Genomic DNA was extracted from whole blood samples using the QIAamp DNA Blood mini kit (Qiagen, Valencia, CA, USA) according to the manufacturer's instructions. Primers were designed using the Assay Designer 3.1 software and were synthesized by the Beijing Genomics Institute, China. The MassARRAY method was used to analyze the genotype of the two selected SNP loci. PCR amplification was performed on the 384 aperture configuration PCR (Applied Biosystems, Beijing, China). The PCR cycling conditions included an initial denaturation step at 94 °C for 5 min, followed by 45 cycles of denaturation at 94 °C for 20 s, annealing at 56 °C for 30 s, and an extension at 72 °C for 1 min, and a final extension step at 72 °C for 3 min. Sequencing of the PCR product was performed with the MassARRAY analyzer (Applied Beijing Genomics Institute, Beijing, China).

### Statistical analyses

Statistical analyses of the data were performed using the SPSS 20.0 software (SPSS Inc., Chicago, IL, USA). By applying the Hardy–Weinberg principle, we examined the group representation of the gene frequencies in each group. The measurement data were assessed using the *t*-test, and the categorical data were assessed using a multilayer Chi-square test or Fisher's exact test. The intergroup allele and genotype frequency comparisons were performed using the chi-square test. The relative risks of the genotypes are indicated by the dominance ratio (Odds Ratio – OR) and the 95% Confidence Interval (95% CI). The difference was statistically significant at *p* < 0.05.

## Results

The speech recognition test range of Group A was 26–120 dB and the average hearing threshold was 50.91 ± 19.34 dB. The respective values for Group B were 26–120 dB and 53.53 ± 18.44 dB. The respective values for Group C were −5 dB to 25 dB and 9.22 ± 6.39 dB. The respective values for Group D were −5 dB to 25 dB and 11.33 ± 6.24 dB. The patients were age- and gender-matched between the hearing loss and control groups for the analyses. There were no significant differences in age and gender between the SHL and control groups.

### Genotyping

The genotype and allele frequency distributions of rs11928865 and rs11920109 were in accordance with the Hardy-Weinberg equilibrium.

Table 1 shows the differences in the rs11928865 (*P*_freq_ = 0.937, OR_unadjusted_ = 1.035, 95% CI = 0.787–1.360) and rs11920109 (*P*_freq_ = 0.815, OR_unadjusted_ = 1.052, 95% CI = 0.815–1.300) genotype and allele frequencies for individuals in the 30–50 years of age SHL and control groups, which did not differ significantly from each other (Table 1).

**Table 1:** Association of the rs11928865 and rs11920109 SNPs in the 30–50 year-old sensorineural hearing loss group and the 30–50 year-old control group.

SNP		Genotype distribution					Allele frequencies				OR_Unadjusted_ (95% CI) *P*_freq_
		Control (*n* = 278)	%	Case (*n* = 467)	%		Control	%	Case	%	
rs11928865	TT	188	67.60	321	68.70	T	455	81.83	769	82.33	1.035 (0.787–1.360)
	AT	79	28.40	127	27.20	A	101	18.17	165	17.67	*P*_freq_ = 0.937
	AA	11	4.00	19	4.10						

rs11920109	CC	90	32.40	154	33.00	C	314	56.47	539	57.71	1.052 (0.815–1.300)
	CT	134	48.20	231	49.50	T	242	43.53	395	42.29	*P*_freq_ = 0.815
	TT	54	19.40	82	17.60						

Table 2 shows the distribution of the rs11928865 genotypes and allele frequencies for individuals in Group B and control groups. The genotype distributions of rs11928865 were significantly different between the groups (*P*_freq_ = 0.001, OR_unadjusted_ = 1.463, 95% CI = 1.136–1.884). However, the genotype distributions of rs11920109 SNP in these groups were not significantly different (*P*_freq_ = 0.349, OR_unadjusted_ = 0.846, 95% CI = 0.712–1.047) (Table 2).

**Table 2:** Association of the rs11928865 and rs11920109 SNPs in the 51–70 year-old sensorineural hearing loss group and the 51–70 year-old control group.

SNP		Genotype distribution					Allele frequencies				OR_Unadjusted_ (95% CI) *P*_freq_
		Control *n* = 477	%	Case *n* = 467	%		Control	%	Case	%	
rs11928865	TT	311	65.20	332	75.60	T	776	81.34	759	86.45	1.463 (1.136–1.884)
	AT	154	32.30	95	21.60	A	178	18.66	119	13.55	*P*_freq_ = 0.001
	AA	12	2.50	12	2.70						

rs11920109	CC	220	46.10	185	42.10	C	638	66.88	558	63.55	0.864 (0.712–1.047)
	CT	198	41.50	188	42.80	T	316	33.12	320	36.45	*P*_freq_ = 0.349
	TT	59	12.40	66	15.00						

Table 3 shows logistic analyses results after the data were adjusted for age and gender. Logistic regression models were constructed to control for potential confounding effects on the GRM7 genotypes, which showed a significant difference in the rs11928865 TT versus AT alleles in the 51–70 year-old SHL and control groups (*p* = 0.000, OR_unadjusted_ = 0.578, 95% CI = 0.429–0.779), whereas the difference in the TT vs. AA alleles was not statistically significant (*p* = 0.260, OR = 0.617, 95% CI = 0.266–1.429) (Table 3).

**Table 3:** Logistic regression model: analysis of the effects of GRM7 SNP rs11928865 genotypes in the 51–70 year-old sensorineural hearing loss groups and the 51–70 year-old control groups.

SNPs	Genotype	Control	Case	*p*	OR	95% CI
rs11928865	TT	311	332			
	AT	154	95	0.000	0.578	0.492–0.779
	AA	12	12	0.260	0.617	0.266–1.429

The genotype distributions were significantly different between individuals aged between 51 and 70 years with or without SHL, indicating that the rs11928865 SNP in GRM7 is associated with the susceptibility to SHL in individuals in this age range. However, rs11928865 was not significantly different between individuals aged between 30 and 50 years with and without SHL, which suggests that rs11928865 is not related to the susceptibility to SHL in this age group. Furthermore, no association was identified between rs11920109 and SHL in this study.

## Discussion

The incidence of SHL is closely related to age, and the incidence of SHL gradually increases with age. Environmental and genetic factors are the two leading causes of SHL. Genetic factors account for 35%–55% of the age-related hearing impairment.^3^ Gene abnormalities such as the encoding connexin 26 gene mutation,^4^ grainyhead-like 2 frame shift mutation,^5^ and cadherin gene mutations have been associated with SHL.^6^ However, most adult SHL lacks a typical family genetic history.

This study shows that rs11928865 in GRM7 was associated with susceptibility to SHL, which is consistent with the results of previous studies.^1^, ^2^ However, the subjects in the previous studies differed with respect to the inclusion and exclusion criteria used in this study as well as other factors such as age, gender, and general condition. Luo et al. selected Chinese males who were aged between 70 and 100 years as their study subjects, and the study of systemic disease was also included in the study.^2^ rs11928865 showed a significant association with SHL in another study with European subjects aged 53 years or older.^1^ In another study, the American subjects had an average age of 71.3 years; a significant correlation between GRM7 SNPs and auditory perception was reported.^7^

Furthermore, previous studies have also demonstrated that gender and age may affect SHL.^8^, ^9^ The risk for SHL directly correlates with age; men are more susceptible to SHL than women, and hearing loss tends to be more severe in men than in women.^10^

In the results of this study, no association between the GRM7 rs11928865 locus and SHL in group A, and no positive results for the rs11920109 locus were observed, reflecting the complexity of SHL due to the involvement of multiple genes, environmental factors, ototoxic drugs, systemic diseases, and lifestyle habits. A number of pathogenic candidate genes including the genes encoding connexin 26 (GJB2),^4^, ^11^, ^12^ grainyhead-like 2 (GRHL2),^5^ cadherin (CDH23),^6^ potassium voltage-gated channel member 4 (KCNQ4),^13^ N-acetyltransferase (NAT),^14^, ^15^, ^16^ apolipoprotein E (APOE),^17^ and uncoupling protein 2 (UCP2)^18^, ^19^ have been related to the genetic susceptibility of SHL, and it is likely that these genes interact in the pathogenesis of SHL.

## Conclusion

In conclusion, rs11928865 was associated with susceptibility to SHL for individuals in group B, but not for individuals in group A. This suggests that differences in the age of onset of SHL in the two age groups, as measured by the time and nature of hearing loss, might be explained by different genetic etiologies.

## Conflicts of interest

The authors declare no conflicts of interest.
